# Effect of Fiber Characteristics on Cracking Resistance Properties of Stone Mastic Asphalt (SMA) Mixture

**DOI:** 10.3390/polym17192623

**Published:** 2025-09-28

**Authors:** Kai Yang, Wenyuan Huang, Mutian Sun, Zhixian Zheng, Hongwei Lin

**Affiliations:** 1 Guangdong Expressway Co., Ltd., Guangzhou 510001, China; yangkai771359968@163.com; 2 Chongqing Kuntong Road and Bridge Design Consulting Co., Ltd., Chongqing 400038, China; hwuyuen@163.com; 3 Key Laboratory of Road and Traffic Engineering of Ministry of Education, Tongji University, Shanghai 201804, China; 2110202@tongji.edu.cn (M.S.); linhw@tongji.edu.cn (H.L.)

**Keywords:** asphalt mixture, fiber modification, crack resistance, mechanical test, microstructural analysis

## Abstract

Cracking is a critical distress that reduces an asphalt pavement’s service life, and fiber reinforcement is an effective strategy to enhance anti-cracking capacity. However, the effects of fiber type, morphology, and length on key cracking modes remain insufficiently understood, limiting rational fiber selection in practice. This study systematically evaluated the influence of four representative fiber types on the anti-cracking performance of Stone Mastic Asphalt (SMA) mixture, combining mechanical testing and microstructural analysis. The fibers included lignin fiber (LF); polyester fiber (PF); chopped basalt fiber (CBF) with lengths of 3 mm, 6 mm, 9 mm; and flocculent basalt fiber (FBF). Key mechanical tests assessed specific cracking behaviors: three-point bending (low-temperature cracking), indirect tensile (tensile cracking), pre-cracked semi-circular bending (crack propagation), overlay (reflective cracking), and four-point bending (fatigue resistance) tests. A scanning electron microscopy (SEM) test characterized fiber morphology and fiber–asphalt interface interactions, revealing microstructural mechanisms underlying performance improvements. The results showed that all fibers improved anti-cracking performance, but their efficacy varied with fiber type, appearance, and length. PF exhibited the best low-temperature cracking resistance, with a 26.8% increase in bending strength and a 16.6% increase in maximum bending strain. For tensile and crack propagation resistance, 6 mm CBF and FBF outperformed the other fibers, with fracture energy increases of up to 53.2% (6 mm CBF) and CT_index_ improvements of 72.8% (FBF). FBF optimized reflective cracking resistance, increasing the loading cycles by 48.0%, while 6 mm CBF achieved the most significant fatigue life improvement (36.9%) by balancing rigidity and deformation. Additionally, SEM analysis confirmed that effective fiber dispersion and strong fiber–asphalt bonding were critical for enhancing stress transfer and inhibiting crack initiation/propagation. These findings provide quantitative insights into the relationship between fiber characteristics (type, morphology, length) and anti-cracking performance, offering practical guidance for rational fiber selection to improve pavement durability.

## 1. Introduction

Asphalt pavements face challenges from various factors during long-term use. The cyclical temperature changes cause thermal expansion and contraction, while repeated traffic loads exert continuous stress impacts [1,2]. Furthermore, environmental factors such as rainwater and ultraviolet rays exacerbate erosion, rendering asphalt mixtures particularly vulnerable to various types of cracking, including low-temperature shrinkage cracks, fatigue cracks, and reflective cracks [3,4]. A study by J. J. Hajek demonstrated that nearly 60% of pavement distresses are closely associated with cracking [5]. Crack generation not only directly weakens the structural load-bearing capacity of the pavement, leading to reduced surface smoothness and affecting driving comfort, but also creates channels for water infiltration. Cracks serve as channels for water infiltration, accelerating moisture penetration into the underlying layers. This process then initiates a series of chain reactions such as subgrade softening and settlement, which significantly shorten the service life of the pavement. Therefore, improving the cracking resistance of asphalt mixtures has become a key technical requirement for prolonging the service life of asphalt pavements and reducing the total life-cycle costs.

Fiber modification technology, a widely used and highly effective method for improving asphalt mixture performance, has attracted significant attention and application in pavement engineering owing to its capacity to enhance mixture performance [6]. The positive effects of fibers stem from their ability to interact with asphalt mixtures in multiple ways. On one hand, fibers can adsorb and stabilize the asphalt, reducing its flow and segregation [7,8]. On the other hand, fibers act as a bridging reinforcement, spanning micro-cracks within the mixture, transferring stress, and preventing further crack propagation [9,10,11]. Furthermore, the incorporation of fibers into asphalt mixtures creates a strong three-dimensional reinforcement network, which enhances the overall structural integrity of the mixture and significantly improves its performance, particularly in terms of crack resistance [12,13]. At present, fibers are available in numerous varieties. Based on different sources and properties, fibers can be primarily categorized into plant fibers, synthetic fibers, and mineral fibers. Lignin fibers (LF), polyester fibers (PF), and basalt fibers (BF) are typical representatives of these three categories [14]. The substantial disparities in chemical composition, microstructure, and physical characteristics across various fibers result in considerable variations in their physical and chemical properties [15]. Such variation leads to differences in the bonding strength with asphalt and the interaction mechanisms, which distinctly affect the high-temperature stability, low-temperature cracking resistance, and fatigue resistance of asphalt mixtures [16].

LFs are widely used in Stone Mastic Asphalt (SMA) mixture due to their strong adsorption ability and cost advantages—this is particularly critical for SMA mixture, as its skeleton-dense structure and high asphalt content require fibers to inhibit binder drain-down during construction. A study conducted by Zhang et al. found that LF can effectively improve the rutting resistance of SMA-16 mixtures, but also reduce fatigue performance and water stability [17]. PFs dissipate externally applied energy through their own plastic deformation. A study by Wu et al. showed that compared to unmodified mixtures, PF-modified asphalt mixtures exhibit enhanced dynamic mechanical properties, with fatigue life increasing by 1.9, 2.9, and 3.6 times at stress ratios of 0.5, 0.4, and 0.3, respectively [18]. BFs demonstrate excellent mechanical properties and high-temperature resistance, enhancing the mixture’s strength, cracking resistance, high-temperature stability, and fatigue performance [19,20]. Moreover, the effectiveness of fiber modification is not only related to fiber type but also influenced by factors such as fiber content, length, and other parameters [21,22,23]. A moderate fiber content can significantly enhance the road performance of the mixture; however, excessive fiber addition may lead to fiber agglomeration, which not only impairs the effectiveness of fibers but also increases material costs. Wu et al. found that 0.4% PF provided the greatest improvement in the low-temperature cracking resistance of asphalt mixtures [24]. Zhang et al. investigated the influence of fiber content on asphalt mixtures, recommending optimal fiber contents of 0.3%, 0.15%, 0.3%, and 0.15% for LF, BF, PF, and polyacrylonitrile fibers, respectively [25]. Furthermore, fibers that are too short fail to form effective crack-bridging within the mixture, while fibers that are too long tend to agglomerate [23]. The morphology of the fibers also influences the bonding between asphalt and fibers by altering the distribution of the asphalt film on the aggregate surface [26]. Therefore, factors such as fiber type, length, morphology, and physical properties are essential for improving the performance of SMA mixtures.

The prevailing criteria for assessing the cracking resistance of asphalt mixtures primarily depend on individual testing methods, such as low-temperature three-point bending (3PB) tests, to characterize their cracking resistance under specific conditions [27]. However, multiple factors in actual pavement service conditions influence the complex process of crack generation and propagation. A single test method is insufficient to fully reflect the anti-cracking performance under different cracking modes. The semi-circular bending (SCB) test effectively evaluates the propagation of pre-existing cracks of the mixture, while the four-point bending (4PB) fatigue test simulates the fatigue cracking process of the pavement under traffic loading [27,28]. The reflective cracking test is more aligned with the actual scenario of crack propagation from the base layer, and the dynamic modulus test offers important information on cracking resistance under various loading conditions [29]. However, there is insufficient emphasis on the integration of several test methods to comprehensively investigate the effects of fiber characteristics on different cracking modes. This gap limits a more profound understanding of the cracking resistance of fiber-modified asphalt mixtures. Nevertheless, most existing studies have been limited to a single fiber type or one mechanical test, which provides only fragmented insights into cracking resistance. To address this gap, the present study integrates multiple fiber characteristics (type, length, and morphology) with five representative cracking resistance tests, thereby offering a systematic and comprehensive evaluation. To further clarify how this study extends prior work, a comparative summary of previous research is presented in Table 1, listing fiber types, test methods, and key findings. This synthesis highlights the fragmented nature of existing studies and demonstrates the novelty of the present integrated approach, which combines multiple fiber parameters and five cracking tests to provide a holistic understanding of fiber-reinforced asphalt mixtures.

This study identified three types of fibers with significant performance disparities: LF (plant fiber), PF (synthetic fiber), and BF (mineral fiber). BFs included 3 mm, 6 mm, 9 mm chopped basalt fibers (CBFs) and flocculent basalt fibers (FBFs). A series of mechanical tests were performed at different temperatures and loading modes to systematically evaluate the effects of fiber type, length, and morphology on the cracking resistance of asphalt mixtures. The findings of this study can promote the efficient application of fiber reinforcement technology in asphalt pavement engineering and provide experimental evidence to effectively enhance the cracking resistance of asphalt pavements.

## 2. Experimental Program

### 2.1. Raw Materials

#### 2.1.1. Asphalt

The asphalt binder adopted in this study was a Styrene–Butadiene–Styrene (SBS) modified asphalt containing 4.5% SBS by weight. A summary of its principal physical properties is provided in Table 2.

#### 2.1.2. Fiber

**Figure 1:**
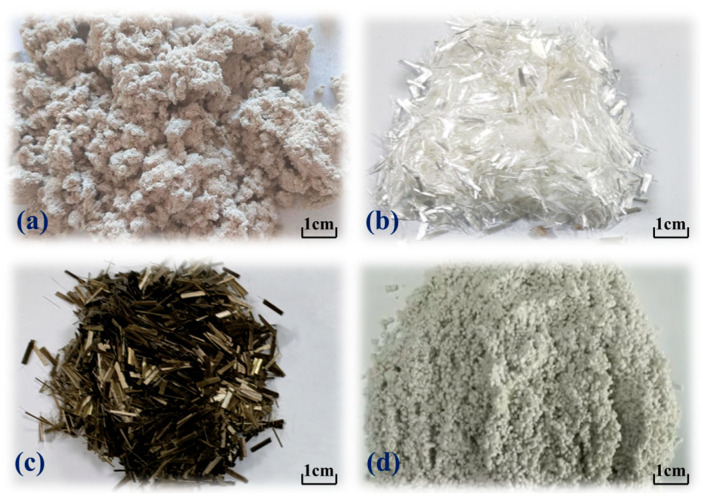
Four types of fibers: (**a**) LF; (**b**) PF; (**c**) CBF; (**d**) FBF.

Four kinds of fibers were incorporated into the experimental design: LF, PF, CBF, and FBF. These fiber types represent plant-based, synthetic, and mineral fibers with distinct reinforcement mechanisms, thereby covering the practical range commonly applied in SMA mixtures. The CBF was further divided into three length groups (3 mm, 6 mm, and 9 mm) to investigate the influence of fiber size on the cracking resistance of asphalt mixtures. This selection was guided by both previous research and practical construction considerations: fibers shorter than 3 mm generally fail to establish effective crack-bridging networks, whereas those longer than 9 mm tend to agglomerate and compromise workability [23]. Detailed technical parameters of the fibers are presented in Table 3, and their physical appearances are illustrated in Figure 1.

### 2.2. Mixtures Design

**Figure 2:**
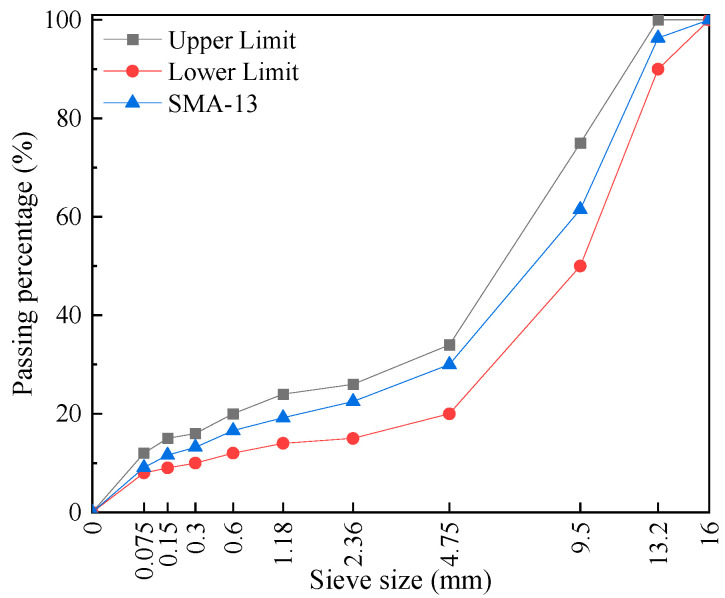
SMA-13 mineral gradation.

Figure 2 shows the aggregate gradation of the SMA-13 mixture. In accordance with the Chinese standard JTG F40-2004 [34], the mix design of SMA-13 mixture was conducted using the volumetric design method for Marshall specimens. Table 4 presents the mix design results of different fiber-modified SMA-13 mixtures, including key parameters such as optimum asphalt content (OAC), voids in the mineral aggregate (VV), voids in the mineral aggregate (VMA), (voids filled with asphalt) VFA, etc. Following the guidelines of Chinese standard JTG F40–2004 [34], fibers were incorporated using the dry process, with a fiber content of 0.3% by total mass of the asphalt mixture. To ensure uniform distribution, fibers were gradually mixed with heated aggregates for 3 min before adding the binder.

It should be noted that the OAC differences (ranging from 5.95% to 6.37% as shown in Table 3) reflect necessary adjustments to meet volumetric requirements (VV, VMA, and drain-down control). While binder content is known to affect cracking indices to some extent, the relatively narrow OAC range observed here suggests that its influence on performance is likely limited compared with the dominant contribution of fiber characteristics [16,35].

### 2.3. Experimental Methods

#### 2.3.1. Experimental Framework

**Figure 3:**
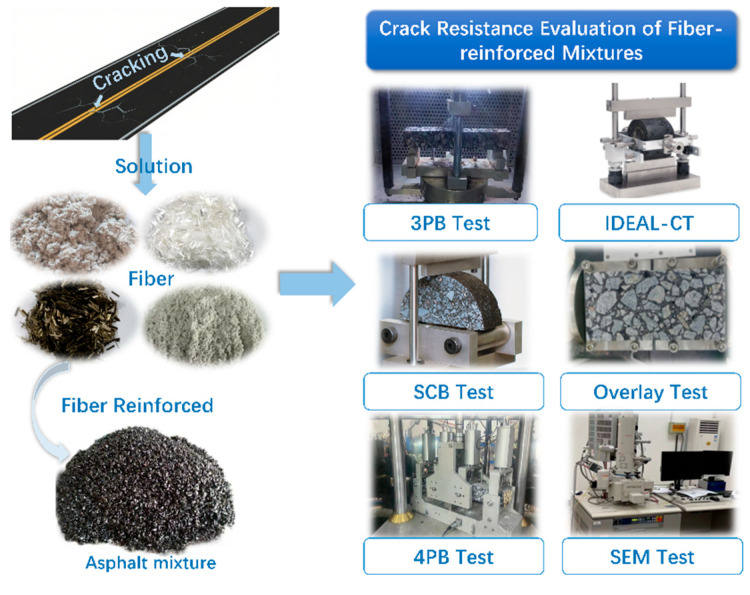
Experimental framework.

This study focuses on evaluating the effects of different types of fibers on the crack resistance of asphalt mixtures, with a systematic investigation into their mechanical responses under varying temperature conditions and cracking modes. The experimental design encompasses key temperature ranges: −10 °C (representing low-temperature cracking), 25 °C (reflecting intermediate-temperature performance), and 15 °C (simulating long-term service fatigue behavior). To address the specific cracking mechanisms at each temperature, distinct test methods were employed. The 3PB test as used to assess low-temperature shrinkage cracking behavior. The indirect tensile cracking test (IDT-CT, formerly called the IDEAL-CT) was conducted to evaluate tensile cracking resistance under the condition of no initial cracks. The pre-cracked SCB test was applied to study the propagation of existing cracks. The overlay test (OT) was utilized to assess anti-reflective cracking performance, while the 4PB fatigue test simulated the fatigue cracking process under cyclic loading conditions. The detailed test procedures are illustrated in Figure 3.

#### 2.3.2. Three-Point Bending Beam Test

The 3PB test was performed to assess the low-temperature cracking resistance of SMA-13 mixtures, in accordance with the Chinese standard JTG F40-2004 [34]. The test procedure followed Section T0715 of the Chinese standard JTG 3410-2025 [27]. Prismatic specimens measuring 250 mm in length, 30 mm in width, and 35 mm in height were used, and the load was applied at a rate of 50 mm/min in a controlled environment at −10 °C. Each test was at least conducted in triplicate to ensure reproducibility and reliability of the results. The primary evaluation parameters are the maximum bending tensile strain (MBS) and the tensile strength at specimen failure, which were calculated using Equations (1) and (2), respectively.

(1) $$R_{B}=\frac{3LP_{B}}{2bh^{2}}$$

(2) $$\varepsilon_{B}=\frac{6hd}{L^{2}}$$

where *P_B_* and *d* represent the maximum load at specimen failure and the mid-span deflection, respectively, while *L*, *b*, and *h* denote the span of the specimen, and the height and width of the middle section, respectively.

#### 2.3.3. Indirect Tensile Cracking Test

The IDEAL-CT method evaluated the cracking behavior of asphalt mixtures under near-pure tensile conditions at ambient temperature [36]. In accordance with ASTM D8225 standard [37], cylindrical specimens with a diameter of 150 mm and a thickness of 62 mm were used. The test was conducted at 25 °C with a loading rate of 50 mm/min. Each test was at least performed in triplicate to ensure reproducibility and reliability of the results. Based on the obtained load–displacement curve, the cracking tolerance index (*CT*_index_) were calculated, as detailed in Equation (3).

(3) $$CT_{\mathrm{index}}=\frac{t}{62}\times \frac{l_{75}}{D}\times \frac{G_{f}}{\left|m_{75}\right|}\times 10^{6}$$

where *D* and *t* denote the diameter and thickness of the specimen, respectively; *l*_75_ is the displacement at 75% of the peak value in the post-peak section; |m_75_| refers to the absolute value of the post-peak slope. The fracture energy, *G_f_*, is calculated as the ratio of the failure work (i.e., the area under the load–displacement curve) to the cross-sectional area of the specimen.

#### 2.3.4. Semi-Circular Bending Test

The SCB test simulates the propagation of pre-existing cracks at the bottom of pavement layers under bending stress [38]. As specified in Section T7044 of Chinese standard JTG 3410-2025 standard [34], the test specimens were pre-cut at the bottom with a 15 mm depth and 1.5 mm width. The test was conducted at 25 °C with a loading rate of 50 mm/min, and at least three parallel tests were conducted for each mixture to ensure the reliability and reproducibility of the results. Key performance indicators, including fracture energy (*G_f_*,) and flexibility index (FI), were calculated using the formulas provided in Equations (4) and (5).

(4) $$G_{f}=\frac{W_{f}}{Area_{lig}}\times 10^{6}$$

(5) $$FI=\frac{G_{f}}{\left|m\right|}\times 0.01$$

where *W_f_* denotes the fracture work, *Area_lig_* represents the fracture area; and |*m*| is the absolute value of the post-peak slope. *G_f_* is the fracture energy, with higher values indicating better crack resistance. *FI* reflects the crack propagation rate, with lower values corresponding to faster crack growth.

#### 2.3.5. Overlay Test

The primary failure mode of reflective cracking involves abnormal increases in local shear stress and strain, whose progression directly affects the structural integrity and service performance of asphalt pavements. The OT method was employed to evaluate the reflective cracking resistance of asphalt mixtures [29]. The specimen dimensions were 150 mm in length, 75 mm in width, and 35 mm in thickness. Horizontal displacement was used as the control parameter, with an opening displacement set to 0.635 mm. A triangular wave cyclic displacement control mode was adopted to simulate the periodic tensile and compressive effects on pavement structures under vehicle loading. The test was conducted at 25 °C with a loading frequency of 0.1 Hz. Each test was at least performed in triplicate to ensure reproducibility. The fatigue life is defined as the number of loading cycles at which the applied tensile force drops to 7% of its initial value. The key parameters are the initial peak load during the first loading cycle and the fatigue life of the specimen.

#### 2.3.6. Four-Point Bending Test

The fatigue cracking resistance of asphalt mixtures is a key indicator to resist cumulative damage under cyclic loading. The 4PB test was employed to effectively simulate the bending deformation and stress conditions of asphalt pavement under vehicle loading. According to Section T0739 of the Chinese standard JTG 3410-2025 [27], prismatic specimens with 380 mm in length, 63.5 mm in width. and 50 mm in height were adopted. The test was conducted at a temperature of 15 °C, with a strain level of 600 με and a loading frequency of 10 Hz. All tests were at least conducted three times to ensure consistent and reliable results. Fatigue failure is determined when the stiffness modulus of the specimen decreases to 50% of its initial value, at which point the load application is terminated.

#### 2.3.7. Scanning Electron Microscope (SEM) Test

The morphology of fibers significantly influences their dispersion within asphalt mixtures and their bonding interaction with the asphalt binder. The microstructure of the asphalt-fiber interface plays a crucial role in determining the fibers’ ability to effectively transfer stress and inhibit the formation and propagation of microcracks within the mixture [39]. A Hitachi SU8010 Cold Field Emission Scanning Electron Microscope (Hitachi High-Technologies Corporation, Tokyo, Japan) was used to capture the micromorphology of various fibers and their interaction with the asphalt binder. The fiber samples examined included LF, PF, FBF, and 6 mm CBF. Due to challenges in distinguishing fibers of different lengths under microscopic conditions, the 6 mm CBF was selected as the representative sample for this study.

## 3. Results and Discussion

### 3.1. Low-Temperature Cracking Resistance

**Figure 4:**
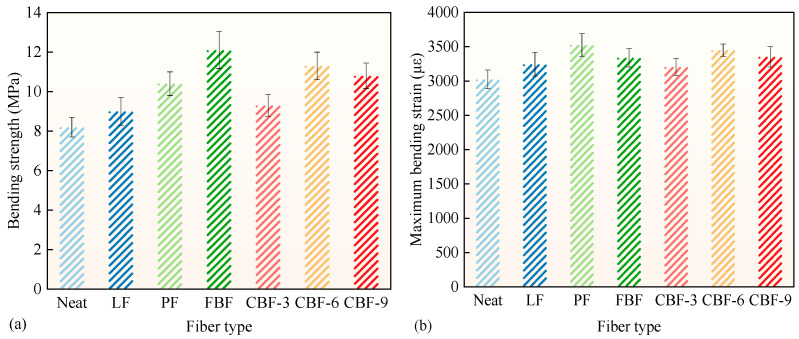
The results of 3PB bending test: (**a**) Bending strength; (**b**) Flexural strain. (Note: Error bars represent the standard deviation of three parallel tests).

The 3PB test was employed to assess the low-temperature cracking resistance of various fiber-modified SMA-13 mixes, as seen in Figure 4. Figure 4 illustrates that the addition of fibers markedly improved the bending performance of the SMA-13 mixture. PF-reinforced SMA-13 exhibited a 26.8% enhancement in bending strength and a 16.6% improvement in MBS value compared to the control group. This enhancement is primarily attributed to PF’s superior elasticity, ductility, and strength, which effectively equilibrate the mixture’s strength and deformation capacity [18]. The incorporation of 3 mm, 6 mm, and 9 mm CBF resulted in enhancements of 13.4%, 37.8%, and 31.7% in bending strength, and 6.0%, 14.1%, and 10.9% in MBS values, respectively [6]. The excellent tensile strength and elasticity modulus of BFs facilitate effective stress distribution, minimizing microcrack development and enhancing resistance to low-temperature cracking [11,30]. The influence of fiber length on low-temperature crack resistance was significant, with the 6 mm CBF demonstrating superior enhancement compared to the 3 mm or 9 mm CBF [22,23].

Furthermore, in comparison to 6 mm CBF, FBF-modified SMA-13 exhibited a 7% increase in bending strength but a 3% decrease in MBS value. This can be attributed to the coarser surface of FBF, which improves adhesion to the asphalt, elevating the stiffness modulus at low temperatures while reducing flexibility [26]. Unlike CBF and PF, LF does not intrinsically enhance cracking resistance; instead, its absorptive properties increase the effective asphalt binder content in the mixture [14]. When properly accounted for in the mix design, this effect indirectly improves performance, leading to 9.8% higher bending strength and 7.3% higher MBS value, which is comparable to that of 3 mm CBF. The Chinese standard JTG F40-2004 recommends MBS as the control index for assessing the low-temperature performance of asphalt mixtures, with PF demonstrating superior improvement in enhancing low-temperature fracture resistance.

### 3.2. Tensile Cracking Resistance

**Figure 5:**
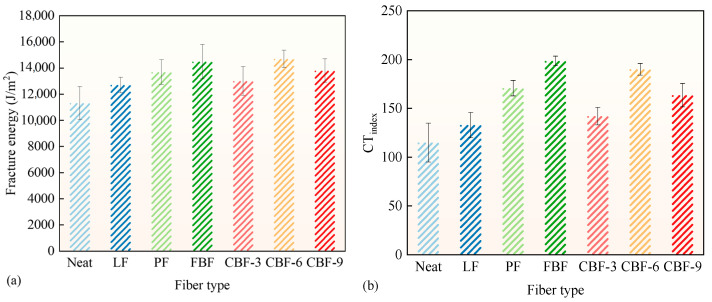
The IDEAL-CT results: (**a**) Fracture energy; (**b**) CTindex. (Note: Error bars represent the standard deviation of three parallel tests).

The IDEAL-CT method was employed to assess the vertical cracking resistance of SMA-13 mixes enhanced with various fibers, as illustrated in Figure 5. Compared to the control group, the fracture energy of SMA-13 modified with 6 mm CBF, FBF, PF, 9 mm CBF, 3 mm CBF, and LF exhibited increases of 29.8%, 27.9%, 20.9%, 21.8%, 14.9%, and 12.2%, respectively. The CT_index_ values rose by 65.3%, 72.8%, 48.5%, 42.3%, 23.6%, and 15.7%, respectively. The findings demonstrate that the incorporation of fibers markedly enhances the tensile cracking resistance of asphalt mixtures.

Among the investigated fibers, 6 mm CBF and FBF showed comparable enhancing effects, both surpassing PF and LF. This is due to the enhanced physical and mechanical capabilities of BFs, which offer greater reinforcement and create a resilient three-dimensional network structure within the mixture [4,40]. This additionally augments the load transfer and absorption capabilities of the mixture, diminishing local stress concentrations and enhancing crack resistance [7]. Conversely, the 3 mm CBF exhibited constrained bridging capacity due to its reduced length. While it can still inhibit crack propagation in the initial stages, its overall efficacy in enhancing cracking resistance was diminished [23]. Likewise, 9 mm CBF, possessing a lower fiber quantity per unit mass, had a diminished reinforcing effect [22]. In conclusion, FBF and 6 mm CBF demonstrated superior efficacy in enhancing vertical cracking resistance.

### 3.3. Crack Propagation Resistance

**Figure 6:**
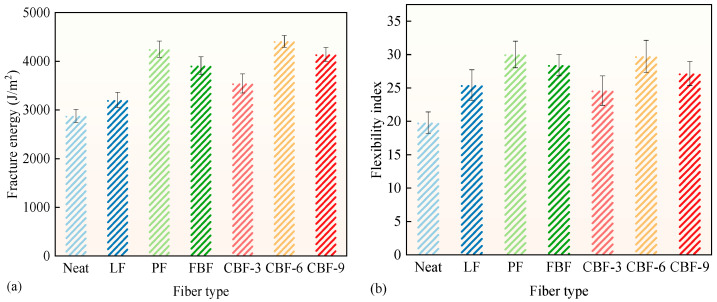
The results of SCB test: (**a**) fracture energy; (**b**) flexibility index. (Note: Error bars represent the standard deviation of three parallel tests).

The pre-cracked SCB test was used to assess the crack propagation resistance of fiber-modified SMA-13 mixes, with findings illustrated in Figure 6. The incorporation of fibers markedly improved the crack propagation resistance of SMA-13 mixes. Compared to the control group, the enhancement in fracture energy for SMA-13 modified with fibers is graded as follows: 6 mm CBF (+53.2%) > PF (+47.6%) > 9 mm CBF (+43.9%) > FBF (+35.8%) > 3 mm CBF (+23.1%) > LF (+11.3%). The ranking of the FI value is as follows: PF (+51.6%) > 6 mm CBF (+50.2%) > FBF (+43.5%) > 9 mm CBF (+37.2%) > LF (+28.4%) > 3 mm CBF (+24.2%). Among these, 6 mm CBF and PF demonstrated the most effective modification effects. The findings demonstrate that moderate length and superior physical properties of fibers provide enhanced stress distribution in stress concentration areas, contributing to elevated fracture energy that bolsters resistance to reflection cracking [18,30,41,42]. FBF enhances resistance to fracture propagation by advantages of greater surface area, excellent adhesion, and superior mechanical qualities, while its inadequate fiber dispersion constrains its overall efficacy. The 9 mm CBF markedly improves the mixture’s rigidity by connecting and creating a fiber-reinforced network. Nonetheless, its effect in improving flexibility is comparatively insignificant. The effectiveness of LF is constrained due to its inferior tensile strength and inadequate dispersion [31]. The limited length of 3 mm CBF constrains its improvement in enhancing crack propagation resistance [42]. In conclusion, 6 mm CBF and PF demonstrate superior efficacy in augmenting fracture propagation resistance.

### 3.4. Reflective Cracking Resistance

**Figure 7:**
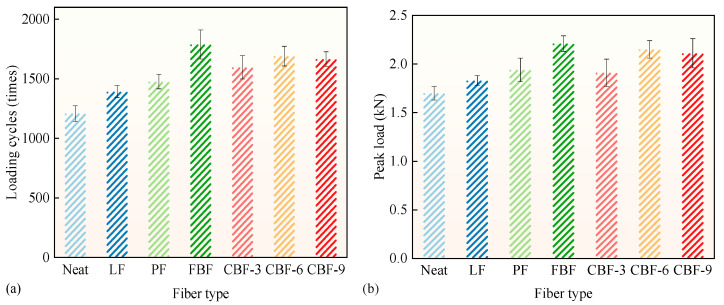
The OT results: (**a**) loading cycles; (**b**) peak load. (Note: Error bars represent the standard deviation of three parallel tests).

The OT method was utilized to assess the resistance to reflective cracking in fiber-modified SMA-13 mixtures, as seen in Figure 7. Figure 7 shows an increase in both the loading cycles and the peak load for the fiber-modified SMA-13 mixtures. In comparison to the control group, the initial peak load for SMA-13 mixtures reinforced by FBF, 6 mm CBF, 9 mm CBF, PF, 3 mm CBF, and LF rose by 30.0%, 26.5%, 24.1%, 14.1%, 12.4%, and 7.6%, respectively. The loading cycles rose by 48.0%, 39.9%, 37.8%, 32.1%, 22.2%, and 15.2%, respectively. Based on these results, the resistance to reflective cracking of the fiber-modified asphalt mixtures can be ranked in descending order as follows: FBF > 6 mm CBF > 9 mm CBF > PF > 3 mm CBF > LF.

The fibers perform as “bridges” inside the asphalt mixture, effectively inhibiting the formation and spread of cracks [43,44]. FBFs have strong bonding strength with the asphalt binder, creating a three-dimensional network that markedly enhances resistance to reflective cracking by promoting uniform cracking resistance and energy dissipation [45]. CBFs strengthen load-bearing capability by effectively transmitting stress, with the 6 mm length offering a good balance between bridging range and dispersion [20]. PF enhances local stress distribution owing to its superior toughness, but LF demonstrates limited improvement due to its inferior strength and inadequate dispersion [46]. FBF demonstrates superior efficacy in resisting reflective cracking.

### 3.5. Fatigue Cracking Resistance

**Figure 8:**
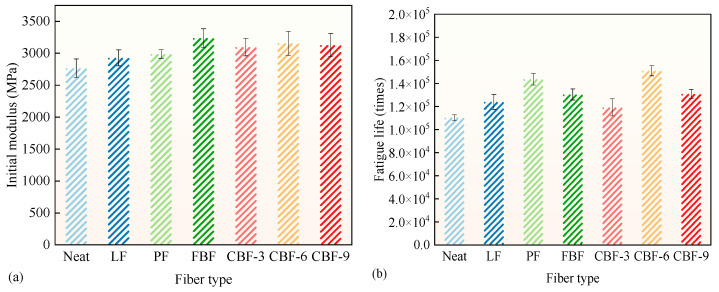
The results of 4PB fatigue test: (**a**) initial modulus; (**b**) fatigue life. (Note: Error bars represent the standard deviation of three parallel tests).

The 4PB fatigue test was utilized to evaluate the fatigue cracking resistance of various fiber-modified SMA-13 mixes, with the findings illustrated in Figure 8. Figure 8a shows that the addition of fibers markedly enhanced the initial stiffness modulus of the SMA-13 mixtures. Compared to the control group, the enhancements in the initial stiffness modulus for fiber-modified SMA-13 mixes followed the following descending order: FBF (+17.1%) > 6 mm CBF (+14.1%) > 9 mm CBF (+13.0%) > 3 mm CBF (+11.9%) > PF (+8.0%) > LF (+5.9%). The modulus of BF-modified SMA-13 mixes exceeded those of LF and PF. In comparison with LF and PF, BFs are high-performance inorganic fibers characterized by superior tensile strength and elastic modulus, accordingly enhancing the overall stiffness of the asphalt mixture.

Figure 8b indicates that the fatigue life of SMA-13 containing 6 mm CBF demonstrated the most significant enhancement, increasing by 36.9%. The appropriate length of the 6 mm CBF effectively bridges cracks, and its excellent mechanical properties inhibit crack development. This provides a relatively ideal balance between rigidity and deformation [20]. The fatigue life of SMA-13 modified with 3 mm CBF and 9 mm CBF was relatively limited, exhibiting enhancements of merely 8.1% and 18.7%, respectively. The results demonstrate that BFs possess an ideal length, with both shorter and longer fibers being less effective in stress distribution and reinforcing during cyclic loading [32]. The substantial rise in the mixture’s rigidity from BFs renders the mixture more susceptible to fatigue failure.

PF-modified SMA-13 exhibited a 30.2% enhancement in fatigue life, ranking slightly lower than the 6 mm CBF. This results from PF’s superior toughness and well-balanced mechanical characteristics, allowing the mixture to achieve an ideal equilibrium between stiffness and deformation. Moreover, FBF and LF enhanced the fatigue life of the mixture by 18.2% and 12.4%, respectively. For FBF, its coarse surface texture and excellent mechanic property promotes stronger adhesion with asphalt, which helps restrain crack propagation. LF’s strong absorption capacity increases the effective asphalt binder content in the mixture, which indirectly improves fatigue resistance [22]. Nevertheless, inadequate fiber dispersion hindered the full realization of their potential in improving fatigue performance [17,26,31,45]. Based on the results, 6 mm CBF is the optimal selection, balancing load-bearing capacity with fatigue durability.

### 3.6. Fiber–Asphalt Interface Morphology

**Figure 9:**
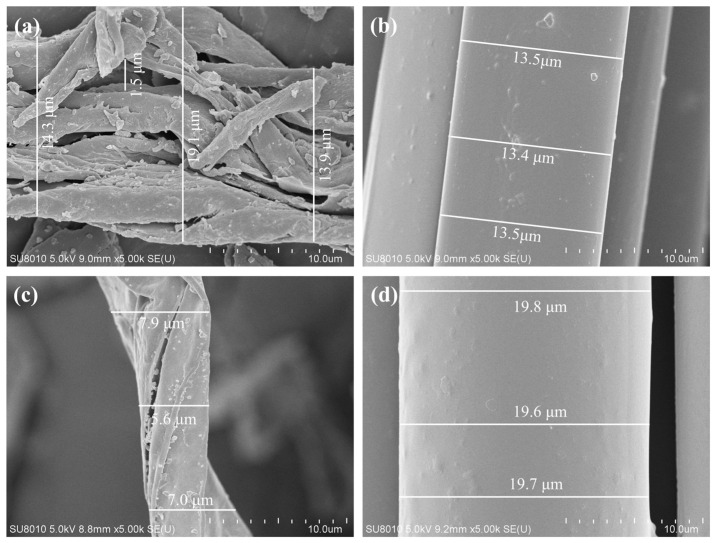
The micromorphology of different fibers: (**a**) LF; (**b**) PF; (**c**) FBF; (**d**) 6 mm CBF.

Figure 9 and Table 5 display the microstructural images of various fibers. Based on SEM morphology and diameter statistics, the fibers can be categorized into two groups: flocculent/branched (LF, FBF) and regular cylindrical (PF, CBF). LF exhibits irregular, in-terlaced fibers with micro-voids, grooves, and hollow features, yielding an average diam-eter of 15.8 ± 2.9 μm (CV ≈ 18.4%). This irregularity enhances asphalt adsorption but compromises tensile uniformity and handling stability [31]. Similarly, FBF shares a flocculent morphology with pronounced branching and irregular thickness, with its diameter of 6.8 ± 1.2 μm (CV ≈ 17.6%), which provides a high surface area for binder adhesion and a three-dimensional mechanical response under external stress [26]. In contrast, PF and CBF present smooth, bundled cylindrical structures with highly uniform diameters (PF: 13.5 ± 0.1 μm, CV ≈ 0.74%; CBF: 19.7 ± 0.1 μm, CV ≈ 0.51%). The regular geometry of PF promotes effective dispersion and predictable reinforcement, while CBF’s larger diameter reduces specific interfacial area yet increases bending stiff-ness, enhancing load-bearing capacity. Collectively, the results indicate that LF and FBF reinforce asphalt mixtures primarily through binder adsorption and spatial interlock, whereas PF and CBF function as geometrically stable linear reinforcements with superior dispersion and tensile consistency.

**Figure 10:**
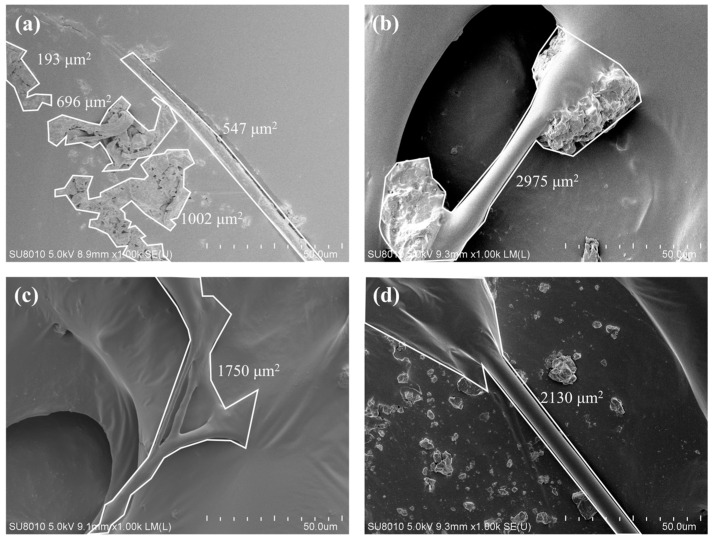
The micromorphology of fiber–asphalt interface area: (**a**) LF; (**b**) PF; (**c**) FBF; (**d**) 6 mm CBF.

Figure 10 displays the microstructural images of the fiber–asphalt interface. As seen in Figure 10a, the interface between LF and asphalt exhibits weak bonding—quantitatively reflected by the smallest bonding area (547 μm^2^) among all fibers—accompanied by a large agglomeration area (1890 μm^2^). This localized aggregation and insufficient interface adhesion restrict LF’s contribution to improving crack resistance and toughness, which explains the relatively modest performance gains observed in our tests [14]. This outcome is consistent with the conventional role of LF in SMA, where it is primarily used to control drain-down rather than to enhance cracking resistance.

Figure 10b shows strong adhesion between PF and asphalt, consistent with its largest bonding area (2975 μm^2^). PF forms a network via bridging, creating a mechanical anchoring effect. The asphalt uniformly coats the fiber surface at the interface, and the absence of agglomeration ensures effective stress transfer. This enhances the overall durability and fatigue resistance of the mixture. The excellent interface adhesion significantly inhibits crack propagation, improving the material’s resistance to reflective cracking [33].

In Figure 10c, the interface between FBF and asphalt displays an increased surface area and a denser coating structure, with a bonding area of 1750 μm^2^. The fibrous flocculence provides numerous contact points, establishing a strong interaction with the asphalt matrix [17]. However, some dispersion issues are observed, which result in poor fiber distribution within the asphalt mixture, negatively impacting its effectiveness in enhancing the mixture’s crack resistance. Despite this, the strong interface adhesion still supports the mixture’s toughness and crack resistance [26].

Figure 10d illustrates a uniform coating of asphalt on the CBF surface, with a bonding area of 2130 μm^2^. This effective coating forms a robust interfacial bond, and CBF’s regular cylindrical structure avoids agglomeration, providing substantial reinforcing effect [20]. This stabilizes the mixture’s internal structure, enhances deformation resistance, and ultimately improves crack resistance [3].

## 4. Conclusions

This study systematically evaluated the influence of different fiber types and lengths on the cracking resistance of SMA-13 mixtures under multiple test conditions, covering low-temperature bending, tensile cracking, crack propagation, reflective cracking, and fatigue performance. The major findings can be summarized as follows:

(1) All fibers significantly improved the low-temperature flexural–tensile performance of SMA-13. PF exhibited the best enhancement, with a 26.8% increase in bending strength and a 16.6% improvement in MBS value. Among CBF lengths, the 6 mm CBF outperformed its 3 mm and 9 mm counterparts.
(2) Fibers significantly enhanced the tensile cracking resistance of SMA-13, as indicated by increased fracture energy and *CT*_index_. 6 mm CBF and FBF showed the most prominent effects, with fracture energy increases of 29.8% and 27.9%, respectively.
(3) The SCB test findings indicated that 6 mm CBF and PF optimized crack propagation resistance, with fracture energy increases of 53.2% and 47.6%, respectively. The moderate fiber length (6 mm) performed better, while inadequate dispersion (for FBF) and suboptimal length (3 mm/9 mm CBF) limited enhancement efficacy.
(4) The OT results demonstrated that FBF provided the best resistance to reflective cracking (30.0% higher peak load and 48.0% more loading cycles), followed by 6 mm CBF.
(5) The 4PB fatigue test further highlighted 6 mm CBF as the best-performing fiber (+36.9% improvement), followed by PF (+30.2% improvement), with other fibers showing moderate or limited improvement.
(6) SEM analysis indicated that fiber morphology and interfacial bonding strongly governed mixture performance. PF and 6 mm CBF exhibited favorable structures for coating and dispersion, while LF and FBF faced challenges due to aggregation or non-uniform distribution.

Overall, the findings highlight that 6 mm CBF and PF provide the best balance of performance enhancement across different cracking modes, while FBF shows particular promise for improving reflective cracking resistance. From an engineering perspective, PF is advantageous for large-scale applications due to its cost-effectiveness and ease of use, whereas 6 mm CBF, though more costly, offers durability and mechanical robustness suitable for high-performance pavements. FBF, despite its strength in mitigating reflective cracking, requires careful attention to mixing and dispersion. Therefore, fiber selection in SMA mixtures should be based not only on mechanical performance but also on cost, availability, and construction feasibility to ensure both effectiveness and practicality in engineering applications. Future work will consider high-binder SMA mixtures without fibers as controls, to better isolate fiber-specific effects and provide deeper insight into their mechanisms, thereby guiding more optimized mixture designs.

## Figures and Tables

**Table 1:** Summary of representative studies on fiber-reinforced asphalt mixtures.

References	Fiber Type	Test Method	Key Findings
Lin et al. [3]	BF	SCB test	Play a role in refining pore distribution while slowing down the spread of micro-cracks.
Song et al. [4]	BF	SCB test	Lower both the crack growth rate and interfacial damage severity, and strengthen resistance to low-temperature cracking.
Wu et al. [7]	BF, PF, and polyacrylonitrile fiber (PAF)	IDEAL-CT/SCB test	The interface adhesion characteristics between fibers and asphalt have a great influence on the crack propagation process.
Phan et al. [9]	Aramid fiber	IDEAL-CT	Enhance the cracking tolerance index and prolong fatigue life.
Hajiloo et al. [10]	Polyolefin–aramid fiber	SCB test	Increases the fracture toughness.
Zhang et al. [11]	BF and LF	IDEAL-CT, 3PB and SCB test	LF and BF can improve the low and intermediate temperature crack resistance of hot recycled mixtures.
Zhang et al. [17]	LF and lignin powder	3PB and 4PB test	Lignin powder-modified asphalt enhances the asphalt mixture’s overall mechanical performance, whereas lignin fiber strengthens thermal cracking resistance but weakens fatigue performance.
Wu et al. [18]	PF	Dynamic modulus test	PF can improve the fatigue performance of asphalt mixtures, especially under lower stress conditions.
Ye et al. [19]	Cellulose, polyester and mineral fiber	Indirect tension fatigue test (ITFT)	Fiber-modified asphalt mixtures showed better fatigue resistance.
Xie et al. [23]	3/6/9 mm BF	BBR	Enhanced cracking potential of asphalt mastics.
Sun et al. [20]	BF	Strip tensile test	BF led to the enhancing effect on toughness capacity of asphalt.
Zhang et al. [25]	LF, BF, PF, and PAF	3PB and 4PB test	Fiber modification enhances the overall performance of the mixture, though the efficacy varies significantly.
Wu et al. [30]	LF and BF	4PB and SCB test	Enhances both the fatigue crack resistance and temperature crack resistance of asphalt mixtures.
Guo et al. [22]	LF, PF and 6/9/15 mm BF	3PB test	6 mm BF enables a significant improvement in the low-temperature cracking resistance of asphalt mixtures.
Pang et al. [31]	Lignin and ceramic fiber (CF)	3PB and 4PB test	SMA mixtures with both fibers had 11% higher low-temperature bending strain and 8% longer fatigue life than those with CF.
Adepu et al. [32]	BF	SCB and ITFT	Mixtures containing 30% RAP and 0.60% basalt fiber exhibited the best fracture and fatigue properties.
Tang et al. [33]	BF, PF, and glass fiber	Disk-shaped compact tension tests	Fiber incorporation enhances asphalt mixtures’ crack resistance; among fibers where BF outperforms PF and glass fibers.

**Table 2:** Principal physical properties of SBS-modified asphalt.

Indices	25 °C Penetration (0.1 mm)	5 °C Ductility (cm)	Softening Point (°C)	135 °C Viscosity (Pa·s)
Value	53.2	35	68	2.6
Standard [34]	40–60	≥20	≥60	≤3

**Table 3:** The physical properties of different fibers.

Indices	Length (mm)	Density (g·cm^−3^)	Elongation at Break (%)	Tensile Strength (MPa)	Elastic Modulus (GPa)
LF	<6	1.10	10–15	<350	3–5
PF	6	1.37	10–25	>800	10–15
FBF	<6	2.77	2–6	2500–3500	100–120
CBF	3/6/9	2.83	>3	>2000	95~110

**Table 4:** The mix design results.

Fiber Type	OAC (%)	VV (%)	VMA (%)	VFA (%)	Theoretical Maximum Specific Gravity	Binder Drainage Loss (%)
Neat	5.95	3.7	18.2	79.4	2.544	0.08
LF	6.37	3.5	17.9	80.6	2.568	0.01
PF	6.18	3.6	18.2	79.7	2.559	0.06
FBF	6.24	3.6	17.7	81.0	2.563	0.02
CBF-3	6.02	3.8	18.4	80.1	2.550	0.05
CBF-6	6.10	3.5	18.3	79.6	2.556	0.06
CBF-9	6.15	3.7	18.4	79.4	2.560	0.06
Standard [34]	/	3–4	≥17	75–85	/	≤0.1%

**Table 5:** The fiber diameter results.

Fiber	Fiber Diameter (μm)					
	①	②	③	Average Value	Standard Deviation	Coefficient of Variation (CV)
LF	14.3	19.1	13.9	15.8	2.9	18.4%
PF	13.5	13.4	13.5	13.5	0.1	0.4%
FBF	7.9	5.6	7.0	6.8	1.2	17.0%
CBF	19.6	19.7	19.8	19.7	0.1	0.5%

## Data Availability

The original contributions presented in this study are included in the article. Further inquiries can be directed to the corresponding author.

## References

[1] Han Z., Pang Y., Lin H. (2025). Improvement of Cold Recycled Mixture Performance Based on Improved Density Test Method and RAP Characteristics. J. Wuhan Univ. Technol.-Mater. Sci. Ed..

[2] Du X., Lin H., Sun M., Liu W., Zhang H. (2024). Field Compaction Characteristics of Ultra-Thin Porous Friction Course Based on Laboratory Simulation. Appl. Sci..

[3] Kong L., Xu J., Yang H., Wu P., Li X., Ma D., Wang Z., Ren D., Ai C. (2025). Enhancing crack resistance in basalt fiber asphalt mixtures: A full-field time-domain investigation of stress distribution and pore structure optimisation. Int. J. Pavement Eng..

[4] Song Y., Sun Y. (2023). Low-Temperature Crack Resistance of Basalt Fiber-Reinforced Phase-Change Asphalt Mixture Based on Digital-Image Correlation Technology. J. Mater. Civ. Eng..

[5] Hajek J.J., HAAS R.C.G. (1987). Factor Analysis of Pavement Distresses for Surface Condition Predictions. Transportation Research Record 1117.

[6] Wu B., Pei Z., Luo C., Xia J., Chen C., Kang A. (2022). Effect of different basalt fibers on the rheological behavior of asphalt mastic. Constr. Build. Mater..

[7] Wu B., Pei Z., Xiao P., Lou K., Wu X. (2022). Influence of fiber-asphalt interface property on crack resistance of asphalt mixture. Case Stud. Constr. Mater..

[8] Chen H., Zhang Y., Bahia H.U. (2021). The role of binders in mixture cracking resistance measured by ideal-CT test. Int. J. Fatigue.

[9] Phan T.M., Nguyen S.N., Seo C.-B., Park D.-W. (2021). Effect of treated fibers on performance of asphalt mixture. Constr. Build. Mater..

[10] Hajiloo H.R., Karimi H.R., Aliha M.R.M., Zanjirani Farahani H., Salehi S.M., Hajiloo M., Jafari Haghighatpour P. (2022). Crack resistance of fiber-reinforced asphalt mixtures: Effect of test specimen and test condition. Fatigue Fract. Eng. Mater. Struct..

[11] Zhang Y., Zhang Y., Li B., Kang A., Wang Y. (2024). Evaluation of Cracking Resistance of SMA-13 Hot Recycling Asphalt Mixtures Reinforced by Basalt Fiber. Materials.

[12] Kaloush K.E., Biligiri K.P., Zeiada W.A., Rodezno M.C., Reed J.X. (2010). Evaluation of Fiber-Reinforced Asphalt Mixtures Using Advanced Material Characterization Tests. J. Test. Eval..

[13] Jia H., Sheng Y., Guo P., Underwood S., Chen H., Kim Y.R., Li Y., Ma Q. (2023). Effect of synthetic fibers on the mechanical performance of asphalt mixture: A review. J. Traffic Transp. Eng. (Engl. Ed.).

[14] Luo D., Khater A., Yue Y., Abdelsalam M., Zhang Z., Li Y., Li J., Iseley D.T. (2019). The performance of asphalt mixtures modified with lignin fiber and glass fiber: A review. Constr. Build. Mater..

[15] Chen H., Xu Q., Chen S., Zhang Z. (2009). Evaluation and design of fiber-reinforced asphalt mixtures. Mater. Des..

[16] Abtahi S.M., Sheikhzadeh M., Hejazi S.M. (2010). Fiber-reinforced asphalt-concrete—A review. Constr. Build. Mater..

[17] Zhang Y., Wang X., Ji G., Fan Z., Guo Y., Gao W., Xin L. (2020). Mechanical Performance Characterization of Lignin-Modified Asphalt Mixture. Appl. Sci..

[18] Wu S., Ye Q., Li N. (2008). Investigation of rheological and fatigue properties of asphalt mixtures containing polyester fibers. Constr. Build. Mater..

[19] Ye Q., Wu S., Li N. (2009). Investigation of the dynamic and fatigue properties of fiber-modified asphalt mixtures. Int. J. Fatigue.

[20] Sun X., Qin X., Chen Q., Ma Q. (2018). Investigation of enhancing effect and mechanism of basalt fiber on toughness of asphalt material. Pet. Sci. Technol..

[21] Gu Q., Kang A., Li B., Xiao P., Ding H. (2022). Effect of fiber characteristic parameters on the high and low temperature rheological properties of basalt fiber modified asphalt mortar. Case Stud. Constr. Mater..

[22] Guo F., Li R., Lu S., Bi Y., He H. (2020). Evaluation of the Effect of Fiber Type, Length, and Content on Asphalt Properties and Asphalt Mixture Performance. Materials.

[23] Xie T., Wang L. (2023). Optimize the design by evaluating the performance of asphalt mastic reinforced with different basalt fiber lengths and contents. Constr. Build. Mater..

[24] Wu J., Hu Y., Jin Q., Ren H. (2024). Macro-microscopic study on the crack resistance of polyester fiber asphalt mixture under dry-wet cycling and neural network prediction. Case Stud. Constr. Mater..

[25] Zhang J., Huang W., Zhang Y., Lv Q., Yan C. (2020). Evaluating four typical fibers used for OGFC mixture modification regarding drainage, raveling, rutting and fatigue resistance. Constr. Build. Mater..

[26] Kou C., Chen Z., Kang A., Zhang M., Wang R. (2022). Rheological behaviors of asphalt binders reinforced by various fibers. Constr. Build. Mater..

[27] (2025). Technical Standards of the Chinese Technical Specifications for Construction of Highway Asphalt Pavements.

[28] (2020). Standard Method of Test for Determining the Fracture Energy of Asphalt Mixtures Using the Semicircular Bend Geometry (SCB).

[29] Zhou F., Hu S., Chen D.-H., Scullion T. (2007). Overlay Tester: Simple Performance Test for Fatigue Cracking. Transp. Res. Rec..

[30] Wu B., Meng W., Xia J., Xiao P. (2022). Influence of Basalt Fibers on the Crack Resistance of Asphalt Mixtures and Mechanism Analysis. Materials.

[31] Pang Y., Li H., Han Z., Wu P., Lin H. (2023). Performance evaluation of asphalt mixture reinforced by lignin and ceramic fiber. J. Eng. Res..

[32] Adepu R., Venkat Ramayya V., Mamatha A., Vinayaka Ram V. (2023). Fracture studies on basalt fiber reinforced asphalt mixtures with reclaimed asphalt pavement derived aggregates and warm mix additives. Constr. Build. Mater..

[33] Tang Q., Xiao P., Lou K., Wu Y. (2024). Interfacial characteristics of fiber asphalt mastic and aggregates: Impact on mixture crack resistance performance. Constr. Build. Mater..

[34] (2004). Technical Specifications for Construction of Highway Asphalt Pavements.

[35] Wu S., Haji A., Adkins I. (2023). State of art review on the incorporation of fibres in asphalt pavements. Road Mater. Pavement Des..

[36] Li H., Xing C., Zhu B., Zhang X., Gao Y., Tang S., Cheng H. (2025). Comparative analysis of four styrene-butadiene-styrene (SBS) structure repair agents in the rejuvenation of aged SBS-modified bitumen. Constr. Build. Mater..

[37] (2019). Standard Test Method for Determination of Cracking Tolerance Index of Asphalt Mixture Using the Indirect Tensile Cracking Test at Intermediate Temperature.

[38] Li M., Han Z., Cheng H., Yang R., Yuan J., Jin T. (2025). Low-temperature performance improvement strategies for high RAP content recycled asphalt mixtures: Focus on RAP gradation variability and mixing process. Fuel.

[39] Xing C., Tang S., Chang Z., Han Z., Li H., Zhu B. (2024). A comprehensive review on the plant-mixed cold recycling technology of emulsified asphalt: Raw materials and factors affecting performances. Constr. Build. Mater..

[40] Seitllari A. (2025). Exploring cracking resistance in modified asphalt mixtures through a comparative assessment of mechanical behavior and performance screening indicators. Constr. Build. Mater..

[41] Wei J., Mao J., Han Y., Li P., Wu W., Yi C. (2025). Influence of Different Fibers on Performance of Bitumen Binders and Thin-Overlay Bitumen Mixtures. Appl. Sci..

[42] Malik M.I., Ahmad A., Mir M.S., ul Haq S.M., Bhat M.M. (2024). Performance assessment of polyester fiber modified stone matrix asphalt mixtures with statistical analysis. Innov. Infrastruct. Solut..

[43] Yucel A.O. (2023). An Evaluation of the Cracking Resistance of Steel- and Glass-Fiber-Reinforced Asphalt Mixtures Produced at Different Temperatures. Sustainability.

[44] Yan J., Du X., Lin H. (2025). Performance Evaluation of Porous Asphalt Mixture Reinforced with Waste Cellulose Acetate Fibers. Sustainability.

[45] Cheng X., Zhong B., Wu Z. (2024). Material-specific coefficients C1 and C2 in Williams-Landel-Ferry equations and phase transition behaviors of asphalt binder reinforced by various basalt fibers. Case Stud. Constr. Mater..

[46] Chen C., Li C., Zhang S., Liu W., Lin H., Zhang H. (2024). Performance Evaluation on Open-Graded Friction Course Reinforced by Double-Adding Fibers Technology. Processes.

